# Theoretical study on electronic and optoelectronic properties of some C^N^N- and C^C^C-chelated iridium(III) complexes for OLEDs

**DOI:** 10.55730/1300-0527.3739

**Published:** 2025-04-15

**Authors:** Ayhan ÜNGÖRDÜ

**Affiliations:** Department of Chemistry, Faculty of Science, Sivas Cumhuriyet University, Sivas, Turkiye

**Keywords:** Iridium(III) complexes, OLEDs, DFT, phosphorescent materials, charge transport properties

## Abstract

The electronic and optoelectronic properties of 8 C^N^N- and C^C^C-chelated Ir(III) complexes were investigated using density functional theory at the Becke-style 3-parameter Lee-Yang-Par and triple zeta plus polarization level. Based on reorganization energy calculations, complex 7 was identified as a promising ambipolar material, while complexes 1 and 2 had efficient hole transport properties. Complex 8 had low ionization potential and is therefore a strong candidate for hole transport applications. Complex 4 had high electron affinity and therefore has potential as an effective electron acceptor material. Photophysical analysis showed that all complexes had phosphorescent properties, with complexes 5 and 6 showing particularly small singlet-triplet energy gaps, making them ideal for high-performance phosphorescent organic light-emitting diodes (PhOLEDs). The intersystem crossing and reverse intersystem crossing rates indicated that these complexes are more likely to have phosphorescence rather than thermally activated delayed fluorescence. These findings provide valuable insights for the design of efficient OLED materials.

## 1. Introduction

Phosphorescent heavy metal complexes, particularly those containing Cu(I), Re(I), Au(I), Os(II), Pt(II), Ru(II), and Ir(III), have been widely used in various applications, including chemosensors, biological imaging, and sensing, owing to their unique photophysical properties [1–4]. Ir(III) complexes have garnered significant attention due to their exceptional characteristics, such as high thermal and photochemical stability, phase homogeneity, and tunable emission colors through ligand modification. These properties make Ir(III) complexes highly promising for use in optoelectronic devices, particularly in organic light-emitting diodes (OLEDs). Ir(III) complexes can facilitate efficient singlet to triplet transitions via strong spin-orbit coupling (SOC), a feature inherent to heavy transition metals like iridium. This results in high phosphorescence efficiency and short triplet emission decay times. The rate of intersystem crossing (ISC) between the lowest excited singlet (S_1_) and triplet (T_1_) states is significantly enhanced (approximately 10^12^ s^−1^), surpassing the radiative emission rate from the singlet state (approximately 10^8^–10^9^ s^−1^). Consequently, the internal quantum efficiency of phosphorescence in Ir(III) complexes can theoretically reach 100%, making them ideal candidates for high-performance OLEDs. However, a critical challenge associated with Ir(III) complexes is phase separation when used in doped devices, which can adversely affect device performance. To address this issue, researchers have focused on increasing the bulkiness of the complexes, thereby improving their dispersibility and emission efficiency. The introduction of bulky ligands has led to the development of high-efficiency Ir(III) complexes that effectively suppress aggregation and enhance material solubility [5–6]. For instance, the incorporation of π-conjugated fluorenyl-substituted triarylamine ligands has been shown to improve performance and solubility [7].

In recent years, computational methods, particularly density functional theory (DFT)-based quantum mechanical simulations, have emerged as powerful tools for material design and screening. Unlike traditional trial and error approaches, computational techniques enable high-throughput screening of materials, allowing researchers to identify promising candidates more efficiently [8–10]. These methods have significantly reduced the labor-intensive aspects of material discovery, enabling the rapid elimination of structures that do not meet predefined performance criteria [11–14]. Computational approaches have been widely adopted across various fields, including optoelectronics, due to their ability to save time and resources [15–16]. Moreover, the accuracy of these methods is often validated by comparing computational predictions with experimental data, providing a reliable platform for predicting the properties of novel synthesized compounds [17–18].

**Figure 1:**
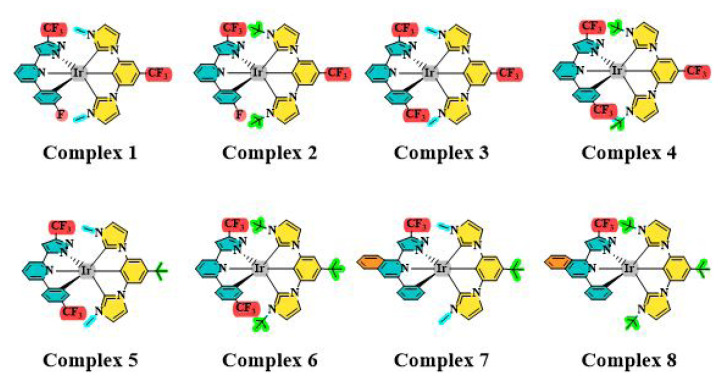
2D representations of the investigated Ir(III)-based complexes [19].

The aim of this study was to predict the OLED-related properties of a series of Ir(III) complexes featuring fluorine-containing bulky ligands (Figure 1) using DFT calculations. By leveraging the theoretical insights obtained, this study aimed to identify and propose suitable Ir(III) complexes for specific functional layers within the OLED structure, thereby contributing to the development of high-performance OLED devices.

## 2. Methods

Computational chemists use many programs to predict the electronic or optical properties of molecules. Amsterdam Modeling Suite (AMS) is considered one of the best computational chemistry software packages for modeling molecular properties. In particular, the Amsterdam density functional (ADF) module of AMS can be used to predict many properties of molecules. All of the calculations reported in this paper were carried out in a vacuum using the ADF module of AMS 2023 [20].

DFT is commonly used for multielectron systems [21, 22] like organic metals. The Becke-style 3-parameter (B3) hybrid functional that incorporates the Lee-Yang-Par (LYP) correlation functional (B3LYP) is a popular hybrid function of DFT [23, 24]. The B3LYP hybrid functional was used in this study to calculate the electronic structure within the framework of DFT. The B3LYP hybrid functional accurately predicts parameters such as molecular orbital energies, energy band gaps, and transition dipole moments that are crucial for understanding the electronic and optical properties of organic-based materials like OLEDs. Additionally, B3LYP is known for its ability to accurately describe electron distribution and charge transfer dynamics in π-conjugated systems, making it a suitable choice for studying OLED materials. Thus, B3LYP was chosen to characterize the OLED compounds examined in this study. The basis set of valence triple zeta plus polarization functions (TZP) have good accuracy and reliability. For that reason, DFT calculations were performed using the B3LYP hybrid functional coupled with the TZP basis set.

Electron and hole transfer are an essential process that control chemical reactions and processes in electronic and optoelectronic devices [25–28]. The electron or hole transfer rate of molecule-based organic materials in the active layers can determine the efficiency of OLED devices. The electron or hole transfer rate (k_e/h_) of materials was calculated by the Marcus formula [29] presented in Equation 1.


(1) $$K_{\frac{e}{h}}=\frac{4\pi^{2}}{4}V_{\frac{e}{h}}^{2}\frac{\text{exp}\left(-\lambda_{\frac{e}{h}}/4k_{B}T\right)}{\sqrt{4\pi \lambda_{\frac{e}{h}}k_{B}T}}$$

Where *h* presents Planck’s constant, *V_e/h_* indicates the electronic coupling for electron or hole transfer, and *λ_e/h_* determines the electron or the hole reorganization energy. *T* indicates the absolute temperature (room temperature, 298.15 K), and *k_B_* stands for the Boltzmann constant.

The electron (*λ_e_*) and hole (*λ_e_*) reorganization energies were obtained via the calculated single point energies as shown in Equations 2 and 3 (supplementary information) [30]. From the calculated single point energies, the adiabatic (IP_a_) and vertical (IP_v_) ionization energy values, the adiabatic (EA_a_) and vertical (EA_v_) electron affinity values, and chemical hardness (η) energy were calculated as shown in Equations 4–8 (supplementary information). Additionally, the electronic couplings (V_e/h_), also called transfer integrals, for electron or hole transfer were computed using Equations 9 and 10 (supplementary information) [31].

The fragment orbital approach was applied in the ADF module of AMS for the calculation of electronic coupling. Therefore, V_e/h_ parameters of dimeric structures were obtained [32].

The communication of the first singlet (S_1_) and triplet (T_1_) excited structures have a substantial impact on the fundamental molecular properties of optoelectronic structures like OLEDs. Therefore, a thorough understanding of the fundamental principles governing the interaction between these spin states is of great importance because it has direct implications for the performance of optoelectronic structures. The phosphorescent or thermally activated delayed fluorescence (TADF) efficiencies of materials could be estimated by interpreting their S_1_↔T_1_ transitions. It is widely acknowledged that compounds with lower singlet-triplet energy splitting (ΔE_ST_) are better able to simplify the ISC and reverse intersystem crossing (RISC). Moreover, smaller singlet-triplet gaps provide higher exciton generation and hence higher OLED performance. The ΔE_ST_ was calculated using Equation 11 [33]:


(11) $$\Delta E_{ST}=E_{S_{1}}-E_{T_{1}}$$

where *E_S_*__1__ and *E_S_*__2__ indicate the total energy of S_1_ and T_1_ structures, respectively. The TADF process has the reverse intersystem transition mechanism of upconversion from T_1_ to S_1_. In addition, it is crucial to understand that the RISC competes with the ISC, the latter being the transition from singlet to triplet states. The phosphorescence or TADF performance of the material was estimated by calculating the intersystem transition rate constant (*k_ISC_*) and the reverse intersystem transition rate constant (*k_RISC_*) using the formulas shown in Equations 12 and 13, respectively.


(12) $$k_{RISC}=\frac{1}{3}\frac{2\pi }{\hbar }|\langle S_{1}|{\hat{H}}_{SO}|T_{1}\rangle {|}^{2}\rho_{FCWD}$$

(13) $$k_{ISC}=3k_{RISC}{\left[exp\;\left(\frac{-\Delta_{ST}}{k_{B}T}\right)\right]}^{-1}$$

In Equations 10 and 11, 〈*S*_1_|*Ĥ_SO_*|*T*_1_〉 and *ρ_FCWD_* denote the S_1_-T_1_ SOC matrix element (SOCME) and the Franck-Condon weighted density of states (FCWD), respectively. For the calculation of the SOCME between the S_1_ and T_1_ states, the optimized excited-state geometry (T_1_-state optimized geometry for RISC and S_1_-state optimized geometry for ISC) obtained at the B3LYP/TZP level from the ADF module of AMS was used. 〈*S*_1_|*Ĥ_SO_*|*T*_1_〉 in Equation 12 can be considered as the probability of transition from T_1_ to S_1_, and *ρ_FCWD_* is the thermokinetic barrier that depends on the mentioned transition. It is easy to describe the thermokinetic barrier using the classical Marcus theory (CMT) equation shown in Equation 14 [34].


(14) $$\rho_{FCWD}^{CMT}=\frac{1}{\sqrt{4\pi \lambda_{M}k_{B}T}}exp\;\left(-\frac{{\left(\Delta E_{ST}+\lambda_{M}\right)}^{2}}{4\lambda_{M}k_{B}T}\right)$$

where *λ_M_* denotes the Marcus reorganization energy that can be obtained from Equation 15.


(15) $$\lambda_{M}=E_{S_{1}/T_{1}}-E_{S_{1}/S_{1}}$$

where *E_S_*__1_/_*_T_*__1__ and *E_S_*__1_/_*_S_*__1__ denote the total energies of excited states obtained using the optimized T_1_ and S_1_ geometries, respectively.

## 3. Results and discussion

### 3.1. Molecular geometry

**Figure 2:**
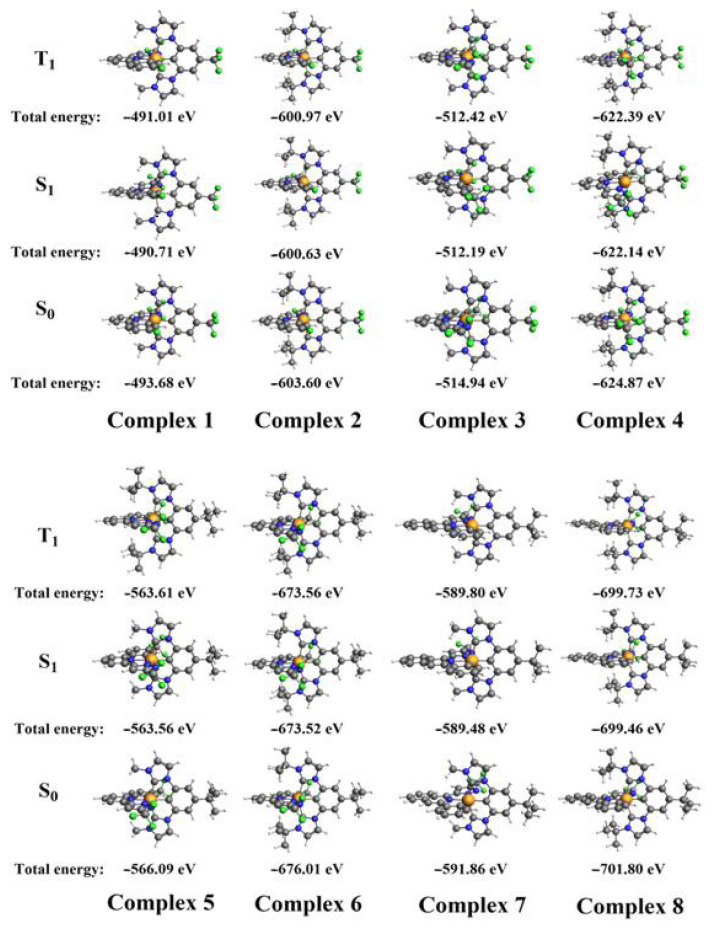
The optimized geometries and total energies of ground and first-excited states for the Ir(III) complexes at B3LYP/TZP level.

The Ir(III) cation adopts a d^6^ electronic configuration [35] that inherently enforces 6-coordination and an octahedral geometry in its complexes [36]. In this study, the ground-state geometries of 8 C^N^N- and C^C^C-chelated Ir(III)-complexes were optimized using the B3LYP hybrid functional and TZP basis set to confirm their structural integrity. As shown in Figure 2, all complexes had 6-coordination around the iridium center, confirming their octahedral geometry. Furthermore, the 2 chelating ligands (C^N^N and C^C^C) were oriented perpendicularly to each other. This geometric arrangement minimizes steric hindrance, thereby enhancing the stability of the complexes. Figure 2 also presents the total energies of the ground and excited states (singlet and triplet) calculated at the B3LYP/TZP level. The results show that the excited-state energies were higher than those of the ground state, consistent with the inherent instability of electronically excited systems. The singlet-state energies were higher than the triplet-state energies.

### 3.2. Frontier molecular orbital analysis

**Figure 3:**
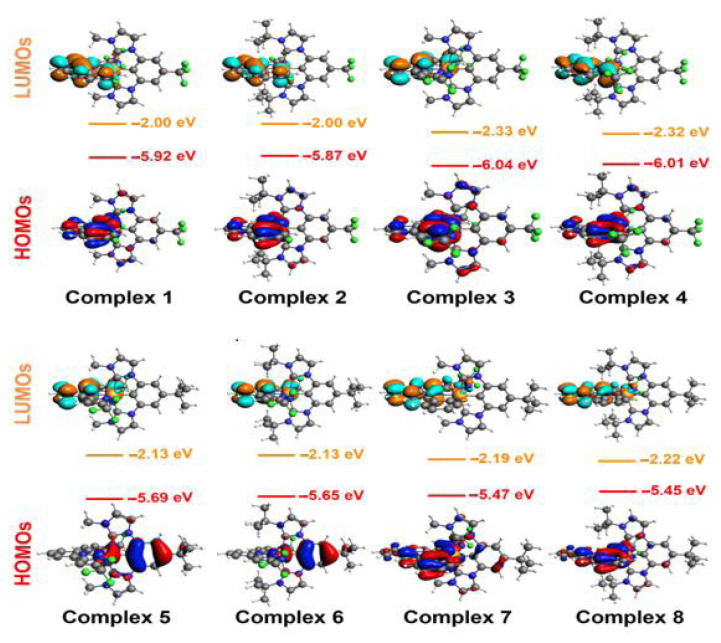
The frontier molecular orbital diagrams and their corresponding energy values for the Ir(III) complexes investigated at the ground state.

Highest occupied molecular orbital (HOMO) and lowest unoccupied molecular orbital (LUMO) are essential parameters for OLED applications. The HOMO can be thought of as the outermost orbital containing electrons, while the LUMO is the innermost orbital ready to accept electrons. In OLED devices, light generation results from the recombination of holes and electrons injected from the electrodes into the emitter layer (EML). The ease of charge injection from the electrodes to adjacent materials in the OLED layers, including the hole injection layer (HIL) and electron injection layer (EIL), is influenced by the HOMO and LUMO levels of these materials. When the HOMO or LUMO energy levels of the materials align with the work function of the adjacent electrode, charge injection improves. This leads to higher charge carrier density and recombination. The HOMO and LUMO energy values of the examined Ir(III) complexes were computed at the B3LYP/TZP level, and the resulting frontier molecular energy levels are shown in Figure 3. The HOMO levels ranged from −6.04 to −5.45 eV, while the LUMO levels ranged from −2.33 to −2.00 eV. For an efficient OLED structure, the work function of the anode adjacent to the HIL layer should be close to these HOMO values. The work function of the cathode adjacent to the EIL layer should be close to these LUMO values. However, charge carrier quenching needs to be considered. The more mobile charge carriers that can reach and become exhausted at the opposite electrode the lower the efficiency of the device. Therefore, it is necessary to prevent these charge carriers from reaching the opposite electrodes. This is achieved using blocking layers, specifically the electron blocking layer (EBL) and hole blocking layer (HBL). In other words, EBL and HBL materials hinder the movement of charge carriers. Modeling the HOMO and LUMO energy levels of materials in these layers is key to achieving this goal. Hole blocking is accomplished by selecting HBL molecules with a high energy gap between the HOMO levels of the EML and HBL materials. This high energy gap acts as a barrier, preventing holes from crossing from the EML to the HBL. Similarly, electron blocking is achieved by choosing EBL compounds with a high energy gap between the LUMO levels of the EML and EBL materials. This high energy gap prevents electrons from passing between the EML and EBL. In both cases, the concentration of charge carriers in the EML increases, enhancing the likelihood of recombination.

The methyl group in complex 1 was replaced with a t-butyl group in complex 2 (Figure 3). Similarly, the same substituent exchange occurred between complexes 3 and 4, 5 and 6, and 7 and 8. The t-butyl group, which releases more electrons than the methyl group, increases the HOMO energy level of the complexes, while it barely changes the LUMO energy level. The fluorine (−F) group in complex 1 was replaced with a trifluoromethyl (−CF_3_) group in complex 3. Similarly, the same change occurs between complexes 2 and 4. The frontier molecular orbital energies given in Figure 3 indicate that the −CF_3_ group, which attracts more electrons than the F group, reduces the HOMO and LUMO energy levels of the complexes.

Interpretation of the distributions of frontier molecular orbitals is useful for OLED studies. The clear separation of HOMO and LUMO distributions reduces the singlet-triplet splitting, resulting in increased phosphorescence and TADF performances. The frontier molecular orbital distributions illustrated in Figure 3 indicate that the HOMO-LUMO separation of complexes 5 and 6 was very good compared to other molecules. The singlet-triplet gap of complexes 5 and 6 was small, facilitating singlet-triplet or triplet-singlet transitions. This means that the phosphorescence and TADF performances of complexes 5 and 6 are expected to be significantly better compared to other molecules examined.

### 3.3. Reorganization energies

Charge transport plays a pivotal role in OLEDs, as light emission originates from the recombination of holes and electrons injected from the anode and cathode into the light-emitting layer. As indicated by the Marcus formula (Equation 1), the reorganization energy (λ_e/h_) is a critical parameter for determining charge carrier mobility. To evaluate the charge transport properties of the studied Ir(III) complexes, the electron (λ_e_) and hole (λ_h_) reorganization energies were calculated at the B3LYP/TZP level. The computed values are summarized in Table 1.

For electron transport, the reorganization energy values of complexes 7 and 8 (λe = 0.27 and 0.25 eV, respectively) are lower than that of the standard electron transport layer (ETL) material, Alq3 (λe = 0.276 eV). This suggests that complexes 7 and 8 are promising candidates for ETL applications. Similarly, for hole transport, complexes 1, 2, 3, and 7 have hole reorganization energies (λ_h_ = 0.18, 0.18, 0.27, and 0.29 eV, respectively) lower than that of the standard hole transport layer (HTL) material, TPD (λ_h_ =0.290 eV). These results highlight the potential of these complexes as HTL materials.

### 3.4. Ionization potentials and electron affinities

Ionization potentials (IPs) and electron affinities (EAs) are quantum chemical descriptors that determine the performance and efficiency of an OLED component. Lower IPs facilitate easier hole formation, leading to improved hole transfer. Thus, compounds with low ionization energy are expected to have high hole transport performance. To estimate the hole transfer efficiency of the studied complexes, adiabatic (IP_a_) and vertical (IP_v_) ionization potentials were calculated at the B3LYP/TZP level, and the computed values are summarized in Table 1. Complex 8 had the lowest ionization energy among the examined complexes, suggesting its potential as an effective hole-transporting material among the investigated complexes. Similarly, higher electron affinity enhances electron acceptance, resulting in more efficient electron transfer. Therefore, molecules with high electron affinity are anticipated to have superior electron transfer efficiency. To evaluate this, adiabatic (EA_a_) and vertical (EA_v_) electron affinities were computed at the same B3LYP/TZP level, with the results presented in Table 1. Complex 4 had the highest electron affinity among the studied molecules, making it a promising candidate for electron acceptor materials in OLED applications.

### 3.5. Chemical hardnesses

Chemical hardness (η), a measure of molecular stability, serves as a reliable indicator for predicting the operational lifetime of OLED components. The calculated η values for the Ir(III) complexes, obtained at the B3LYP/TZP level, are summarized in Table 1. Among the investigated compounds, complex 1 had the highest chemical hardness (η = 3.02 eV), suggesting superior stability and the longest operational lifetime in OLED applications.

### 3.6. Charge transfer integrals

**Figure 4:**
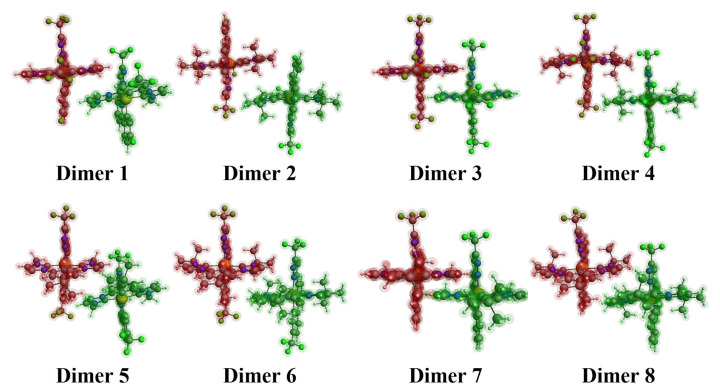
The dimeric forms of the Ir(III) complexes examined at the B3LYP/TZP level.

Accurate calculation of charge transfer rates via the Marcus equation at a given temperature requires the determination of transfer integrals. These integrals, which quantify the electronic coupling between molecules, are typically calculated for dimeric structures. While experimental crystal data are often used to model realistic dimers, charge transfer integrals can also be approximated for hypothetical dimeric geometries without such data. In this study, the investigated complexes were geometrically optimized to form stable dimeric configurations (Figure 4). The electron (V_e_) and hole (V_h_) transfer integrals for these dimers were computed at the B3LYP/TZP level, and the results are summarized in Table 2. Complex 7 had the highest value among the studied complexes, indicating a superior electron transfer rate in the dimeric geometry shown in Figure 4. Similarly, analysis of the hole transfer integrals showed that complexes 3 and 7 possess the largest values, suggesting these materials have enhanced hole transfer capabilities compared to the other complexes. These findings position complexes 3 and 7 as promising candidates for optimizing charge transport in OLED applications.

### 3.7. Charge transfer rates

Once the charge transfer integrals were determined, charge transfer rates could be calculated using the Marcus equation (Equation 1) that incorporates reorganization energies (Table 1) and transfer integrals (Table 2). At room temperature, substituting these values into Equation 1 yields the electron (k_e_) and hole (k_h_) transfer rates, as summarized in Table 2. Analysis of the data showed that complex 7 had the highest electron transfer rate (k_e_) among the studied complexes. Furthermore, Table 2 shows that complex 7 also possesses the highest hole transfer rate (k_h_). This means that complex 7 has potential use as an ambipolar material since it has the highest electron and hole transfer rates among the dimeric structures in Figure 4.

### 3.8. Phosphorescent properties

The ground and excited state geometries of the studied complexes and their corresponding energies were obtained at the B3LYP/TZP level. Using these energies, S_1_-S_0_, T_1_-S_0_, and S_1_-T_1_ energy gaps were calculated and these values are given in Table 3. The S_1_→S_0_ and T_1_→S_0_ transitions corresponds to fluorescence and phosphorescence, respectively. The energy gap values were converted to wavelengths and the calculated emission (fluorescence and phosphorescence) wavelengths are also presented in Table 3. The experimental emission values of the complexes examined are in agreement with the calculated phosphorescence values. This means that all the compounds investigated, including iridium compounds with no experimental emission value, could have phosphorescent properties.

In phosphorescence, ISC is a crucial step in generating triplet exciton. To achieve efficient ISC, large SOC and an optimal singlet-triplet gap are necessary. In small systems with low-lying triplets that are thermally accessible, the ISC and RISC compete against each other. The ISC between singlet and triplet excited states can be greatly improved by a transition metal atom such as Ir(III) that causes a strong SOC effect. Therefore, Ir(III)-complexes are expected to be used as phosphorescent or TADF materials thanks to their ability to convert both singlet and triplet excitons into light via ISC or RISC. In order to predict the phosphorescence and TADF properties of the complexes investigated, all necessary parameters were obtained at the B3LYP/TZP level and these parameters are tabulated in Table 3. On the other hand, the ISC (k_ISC_) and RISC (k_RISC_) rates were obtained using the first singlet-first triplet energy gap, SOCME, and Marcus reorganization energy values of the Ir(III)-compounds examined at room temperature. All k_ISC_ values were higher than k_RISC_ values. This means that the phosphorescence properties of the studied complexes could predominate rather than TADF.

## 4. Conclusions

In this study, the electronic and optoelectronic properties of 8 C^N^N- and C^C^C-chelated Ir(III) complexes were systematically investigated using DFT. Based on the concept of reorganization energy, complex 8 is the most effective electron transfer material, while complexes 1 and 2 are the most efficient hole transfer materials among the studied complexes. Additionally, complex 7 has potential as ambipolar material. Based on IP and EA values, complexes 8 and 4 could be used as good hole transport and electron acceptor materials, respectively. In addition, by comparing the experimental emission values with the calculated emission values, the investigated complexes could have phosphorescence properties. From ISC and RISC rate constants, the studied Ir(III) complexes could show phosphorescence behavior rather than TADF.

## Supplementary information

### Equations


(2) $$\lambda_{e}=(E_{0}^{-}-E_{-}^{-})-(E_{-}^{0}-E_{0}^{0})$$

(3) $$\lambda_{h}=(E_{0}^{+}-E_{+}^{+})-(E_{+}^{0}-E_{0}^{0})$$

(4) $$IP_{a}=E_{+}^{+}-E_{0}^{0}$$

(5) $$IP_{v}=E_{0}^{+}-E_{0}^{0}$$

(6) $$EA_{a}=E_{0}^{0}-E_{-}^{-}$$

(7) $$EA_{v}=E_{0}^{0}-E_{0}^{-}$$

(8) $$\eta =\frac{IP_{a}-EA_{a}}{2}=\frac{E_{+}^{+}+E_{-}^{-}-2E_{0}^{0}}{2}$$

where $E_{0}^{0},E_{-}^{-}$, and $E_{+}^{+}$ values are the single point energies of the optimized neutral, anionic, and cationic forms, respectively. $E_{0}^{-}$ and $E_{0}^{+}$ indicate the single point energies obtained by adding or subtracting one electron in the neutral geometries optimized, respectively. Lastly, $E_{0}^{-}$ and $E_{+}^{0}$ values denote the single point energy values achieved by subtracting or adding an electron in the optimized anionic and cationic structures, respectively.


(9) $${\text{V}}_{\frac{e}{h}}=\frac{{\text{J}}_{ab}-\frac{{\text{S}}_{ab}(E_{a}+E_{b})}{2}}{1-S_{ab}^{2}}$$

In above Equation 9,*E_a_* and *E_b_* parameters represent the site energy values of monomers a and b, respectively. Furthermore, *J_ab_* and *S_ab_* values show the charge transfer and the overlap integrals, respectively. It is possible to express the values of *J_ab_*, *S_ab_*, *E_a_*, and *E_b_* in Equation 10.


(10) $$\begin{array}{l} {\text{J}}_{ab}=\langle \varphi_{a}^{\frac{H}{L}}|h_{KS}|\varphi_{b}^{\frac{H}{L}}\rangle \\ {\text{S}}_{ab}=\langle \varphi_{a}^{\frac{H}{L}}|\varphi_{b}^{\frac{H}{L}}\rangle \\ {\text{E}}_{a}=\langle \varphi_{a}^{\frac{H}{L}}|h_{KS}|\varphi_{a}^{\frac{H}{L}}\rangle \\ {\text{E}}_{b}=\langle \varphi_{b}^{\frac{H}{L}}|h_{KS}|\varphi_{b}^{\frac{H}{L}}\rangle \end{array}$$

Here, *h_KS_* determines the Kohn-Sham Hamiltonian of the dimeric forms. Moreover, $\varphi_{a}^{\frac{H}{L}}$ and $\varphi_{b}^{\frac{H}{L}}$ represent the frontier molecular orbitals (HOMOs or LUMOs) for the 2 monomer forms [32]. It should be noted that hole transport is based on the HOMO of monomers as a basic function, while electron transport is based on the LUMO of monomers.

## Figures and Tables

**Table 1:** The calculated reorganization, ionization, electron affinity, and chemical hardness energies (in eV) for the examined Ir(III) complexes at the B3LYP/TZP level.

Compound	λ_e_	λ_h_	IP_a_	IP_v_	EA_a_	EA_v_	η
**1**	0.36	0.18	6.74	6.84	0.70	0.54	3.02
**2**	0.35	0.18	6.65	6.74	0.71	0.56	2.97
**3**	0.46	0.27	6.82	6.93	1.07	0.87	2.88
**4**	0.44	0.30	6.70	6.83	1.08	0.88	2.81
**5**	0.45	0.35	6.43	6.59	0.88	0.68	2.77
**6**	0.43	0.37	6.32	6.50	0.89	0.70	2.71
**7**	0.27	0.29	6.18	6.32	0.92	0.79	2.63
**8**	0.25	0.31	6.10	6.24	0.93	0.81	2.59

**Table 2:** The achieved electron and hole transfer integral values^a^ (in eV) and the charge transfer rates (s^−1^) for the Ir(III) dimers studied at the B3LYP/TZP level.

Dimer	V_e_	V_h_	k_e_	k_h_
**1**	0.00182	0.01624	2.79 × 10^9^	1.81 × 10^12^
**2**	0.00257	0.00280	6.22 × 10^9^	5.39 × 10^10^
**3**	0.00197	0.07290	1.09 × 10^9^	1.24 × 10^13^
**4**	0.00245	0.00057	2.10 × 10^9^	5.38 × 10^8^
**5**	0.00165	0.00385	8.55 × 10^8^	1.39 × 10^10^
**6**	0.00541	0.00298	1.14 × 10^10^	6.69 × 10^9^
**7**	0.00717	0.09090	1.20 × 10^11^	1.53 × 10^13^
**8**	0.00325	0.01108	3.11 × 10^10^	1.81 × 10^11^

a The transfer integrals were calculated at room temperature by taking the Boltzmann constant as 8.6173 × 10^−5^ eV K^−1^ and the Planck constant as 4.1357 × 10^−15^ eV s.

**Table 3:** The obtained S_1_-S_0_, T_1_-S_0_, ΔE_ST_, fluorescence, phosphorescence, SOCME, Marcus reorganization energy, K_ISC_, and K_RISC_ values for the Ir(III) complexes studied at room temperature.

Complex	S_1_-S_0_^a^	T_1_-S_0_^a^	S_1_-T_1_^a^	Fl.^b^	Ph. ^b^	Exp.^c^	SOCME^d^ S_1_↔T_1_	λ_Marcus_^a^	k_ISC_ S_1_→T_1_	k_RISC_ T_1_→S_1_
**1**	2.97	2.67	0.31	417	465	467	39.57	0.25	4.57 × 10^19^	8.77 × 10^13^
**2**	2.98	2.64	0.34	416	470	-	36.47	0.18	1.32 × 10^19^	7.87 × 10^12^
**3**	2.75	2.52	0.23	450	492	490	76.63	0.18	2.03 × 10^20^	8.77 × 10^15^
**4**	2.72	2.48	0.24	455	500	-	88.23	0.18	2.54 × 10^20^	7.42 × 10^15^
**5**	2.53	2.48	0.05	490	500	501	320.6	0.17	1.84 × 10^21^	8.75 × 10^19^
**6**	2.49	2.45	0.04	498	505	-	506.2	0.24	1.74 × 10^21^	1.22 × 10^20^
**7**	2.39	2.07	0.32	520	599	593	175.47	0.29	9.32 × 10^20^	1.21 × 10^15^
**8**	2.34	2.06	0.27	531	601	-	145.66	0.32	5.84 × 10^20^	5.31 × 10^15^

The units of a and b (or c) are presented as eV and nm, respectively. It should also be noted that c is achieved on the host [37]. The units of d and k_ısc_ (or k_RISC_) is cm^−1^ are given as cm^−1^ and s^−1^, respectively.
